# Evaluation of Antihypertensive Medication Adherence and Its Association With Blood Pressure Control Among Hypertensive Patients Attending Outpatient Clinics in Khyber Pakhtunkhwa, Pakistan: A Cross-Sectional Study

**DOI:** 10.7759/cureus.111179

**Published:** 2026-06-19

**Authors:** Muhammad Adeel Zafar, Abubakar Ahmad, Abbas Uddin, Imadud Din, Savaid Ali, Iman Masood, Anielka Cristina C Bonilla Arauz, Arish Noor

**Affiliations:** 1 Acute Medicine, Royal Glamorgan Hospital, Llantrisant, GBR; 2 Acute Medicine, Sameen Zafar Medical Center, Multan, PAK; 3 Internal Medicine, District Headquarters Teaching Hospital, Dera Ismail Khan, PAK; 4 Internal Medicine, Khyber Medical University, Peshawar, PAK; 5 Pediatrics, Medical Teaching Institution, Ayub Teaching Hospital, Abbottabad, PAK; 6 Internal Medicine, Gomal Medical College, Dera Ismail Khan, PAK; 7 General Medicine, National Autonomous University of Nicaragua, Leon, NIC; 8 Medicine, Khyber Medical College, Peshawar, PAK

**Keywords:** blood pressure control, bmq, hypertension, medication adherence, pakistan

## Abstract

Background: Hypertension is a major noncommunicable disease in Pakistan, and suboptimal blood pressure (BP) control remains a significant public health challenge, largely influenced by poor adherence to antihypertensive medications.

Objective: This study aimed to assess the prevalence of antihypertensive medication adherence and its association with BP control among hypertensive patients attending outpatient clinics in Khyber Pakhtunkhwa, Pakistan.

Methodology: This analytical cross-sectional study included 436 hypertensive patients selected through consecutive sampling from tertiary care hospitals. Data were collected using a structured questionnaire and the validated Brief Medication Questionnaire. BP was measured using standardized procedures with a Medico Aneroid Sphygmomanometer AND-85 and the Omron M3 Comfort device (Omron Healthcare, Kyoto, Japan). BP control was defined as <140/90 mmHg. Data were analyzed using Statistical Package for the Social Sciences version 25 (IBM Corp., Armonk, NY). The chi-square test and multivariable binary logistic regression were used to assess associations and adjust for confounders.

Results: Of 436 participants, 196 (44.95%) were adherent and 240 (55.05%) were nonadherent. BP was controlled in 178 (40.83%) patients and uncontrolled in 258 (59.17%). Controlled BP was significantly higher among adherent patients (67.35%) compared with nonadherent patients (19.17%) (P < 0.001). Nonadherence was independently associated with uncontrolled hypertension (adjusted odds ratio = 3.82; 95% confidence interval: 2.45-5.97; P < 0.001). Other significant predictors included age ≥60 years, diabetes mellitus, and longer duration of hypertension.

Conclusion: Medication nonadherence is a major independent and modifiable determinant of poor BP control among hypertensive patients in Khyber Pakhtunkhwa. Targeted adherence-enhancing interventions are urgently needed in outpatient clinical settings.

## Introduction

Hypertension is one of the most common noncommunicable diseases in the world and a leading cause of cardiovascular morbidity and mortality [1]. It is believed that more than 1 billion people are affected globally, with the highest numbers in low- and middle-income countries [2]. Sustained high blood pressure (BP) is closely connected with negative consequences such as stroke, ischemic heart disease, heart failure, and chronic kidney disease [3]. Although effective antihypertensive therapies are available, BP control remains suboptimal in many populations, particularly in low- and middle-income countries and developing regions [4].

In Pakistan, hypertension is a major public health issue with reported prevalence of between 26% and 33% among adults, and control rates are dismal, with reported control rates at less than 20% [5,6]. This has been driven by rapid urbanization, sedentary lifestyles, unhealthy eating habits, and rising life expectancy [7]. Khyber Pakhtunkhwa, with a mixed urban-rural population and diverse socioeconomic conditions, has other barriers, including limited access to healthcare facilities, low health literacy, financial constraints, and low practices in follow-up. All of these lead to suboptimal disease management, and the risk of complications increases [8].

Medication adherence is widely recognized as a key modifiable determinant of BP control and cardiovascular risk reduction in hypertensive patients [9]. It is defined as the extent to which a patient’s medication-taking behavior corresponds with the agreed recommendations from a healthcare provider [10]. Nonadherence is a multifactorial phenomenon influenced by patient-related, therapy-related, socioeconomic, and healthcare system factors. These include complex drug regimens, adverse drug effects, financial constraints, forgetfulness, low health literacy, limited access to healthcare, and inadequate understanding of the chronic nature and complications of hypertension [11]. Importantly, both intentional and unintentional nonadherence contribute significantly to suboptimal treatment outcomes. Evidence from prior studies indicates that poor adherence remains one of the most consistent and strongest predictors of uncontrolled hypertension despite availability of effective antihypertensive therapies [12].

Hypertension management in outpatient clinics represents the cornerstone of long-term care, follow-up, and treatment adjustment for patients. However, in routine clinical practice, medication adherence is often not systematically assessed, leading to its underrecognition as a major contributor to uncontrolled BP. Furthermore, most existing evidence on adherence patterns and their impact on BP control originates from high-income or mixed settings, with limited context-specific data from resource-constrained regions such as Khyber Pakhtunkhwa, Pakistan. Local healthcare challenges, including variability in access to care, socioeconomic disparities, and differences in patient education levels, may significantly influence adherence behaviors and clinical outcomes. In addition, BP control in the present study was defined as <140/90 mmHg, consistent with WHO and widely adopted international and regional hypertension management recommendations and to facilitate comparison with previous studies conducted in similar healthcare settings [13].

Furthermore, most existing evidence on adherence patterns and their impact on BP control originates from high-income or mixed settings, with limited context-specific data from resource-constrained regions such as Khyber Pakhtunkhwa, Pakistan. Local healthcare challenges, including variability in access to care, socioeconomic disparities, and differences in patient education levels, may significantly influence adherence behaviors and clinical outcomes. In addition, BP control in the present study was defined as <140/90 mmHg, consistent with widely adopted international and regional hypertension management recommendations and to facilitate comparison with previous studies conducted in similar healthcare settings.

## Materials and methods

Study design

This was conducted as an analytical cross-sectional study to assess the association (not causation) between drug adherence and BP control among hypertensive patients. The study aimed to identify statistical relationships between exposure (medication adherence) and outcome (BP control) without inferring temporal or causal effects due to its cross-sectional nature. This study was conducted and reported in accordance with the Strengthening the Reporting of Observational Studies in Epidemiology guidelines for cross-sectional studies.

Study setting

This was conducted in the outpatient units of the Khyber Teaching Hospital, Peshawar, and the Ayub Teaching Hospital, Abbottabad. These tertiary care hospitals provide services to a large heterogeneous population across Khyber Pakhtunkhwa, thus increasing the representativeness and external validity of the study findings. Both hospitals serve as major referral centers, managing patients from urban and rural catchment areas, which allows inclusion of a diverse socioeconomic and clinical population.

Study duration

The study was conducted over a period of one year, from February 2024 to January 2025.

Study population

The study population consisted of adult patients diagnosed with hypertension who attended outpatient clinics of the sampled hospitals and were receiving antihypertensive therapy for at least three months.

Sample size determination

The sample size was calculated using the single population proportion formula: $n = \frac{Z^2 \, P (1 - P)}{d^2}$ [14]. Here Z = 1.96 at a 95% confidence level, P = 0.50 (assumed prevalence of antihypertensive medication adherence due to limited regional data), and d = 0.05 margin of error. The calculated sample size was 384 participants. To compensate for potential nonresponse and incomplete questionnaires, 10% was added, resulting in a final target sample size of 422 participants. All 436 participants had complete datasets with no missing values in key study variables; therefore, all were included in the final analysis.

Sampling technique

A nonprobability consecutive sampling method was used, in which all eligible patients presenting during the study period were recruited until the required sample size was reached. Patients were screened consecutively on each outpatient clinic day by trained data collectors. All eligible patients presenting during the study period were approached for participation. Recruitment continued until the required sample size was achieved. Patients declining participation were recorded as nonresponders but were not replaced selectively. This method was chosen to minimize selection bias by including all available eligible participants in a sequential manner.

Inclusion and exclusion criteria

Patients aged 18 years and above with a confirmed diagnosis of primary hypertension and who had been on antihypertensive medication for at least three months were included. Patients with secondary hypertension, pregnant women, critically ill individuals, and those with cognitive impairment preventing reliable responses were excluded.

Data were checked for completeness before analysis. Questionnaires with missing or incomplete information on key study variables (medication adherence and BP measurements) were excluded. Therefore, a complete-case analysis approach was used to ensure validity of statistical inferences.

Data collection procedure

A structured and pretested questionnaire was used to collect data through face-to-face interviews (see the Appendix). The questionnaire was developed in English and administered through face-to-face interviews by trained healthcare personnel. Prior to study initiation, the questionnaire was pilot-tested with 20 hypertensive patients in a comparable outpatient setting to assess its clarity, comprehension, and feasibility. Data from the pilot phase were not included in the final analysis. After obtaining informed consent, participants were interviewed regarding sociodemographic factors, clinical history, and medication-taking behavior.

BP was measured in a standardized manner using a Medico Aneroid Sphygmomanometer AND-85 set (including a calibrated aneroid manometer, inflatable cuff, and inflation bulb system) and an Omron M3 Comfort automated digital BP monitor (Omron Healthcare, Kyoto, Japan) with an appropriate arm cuff, ensuring consistent and reliable measurement across participants. Each participant was assessed using both devices to cross-verify readings and minimize measurement bias and improve accuracy.

Two BP readings were taken after five minutes of rest in a seated position using the appropriate cuff size, and the average of the two readings was recorded to minimize measurement bias and improve accuracy. Participants were instructed to avoid smoking, caffeine consumption, and vigorous physical activity for at least 30 minutes prior to measurement whenever feasible. Measurements were performed in a seated position with back support and feet resting on the floor after a minimum five-minute rest period. Appropriate cuff sizes were selected according to arm circumference. All devices were calibrated according to manufacturer recommendations prior to data collection to ensure measurement validity and reliability. Measurements were taken at the same time of day whenever feasible to reduce diurnal variation in BP readings.

Assessment of drug adherence

Medication adherence was assessed using the Brief Medication Questionnaire (BMQ), a validated and widely used instrument for evaluating medication-taking behavior and identifying barriers to adherence [15]. The BMQ evaluates adherence across three domains: regimen screen (missed doses), belief screen (perceived effectiveness and concerns), and recall screen (forgetfulness and regimen complexity).

The BMQ consists of these three domains, and participants reporting missed doses, medication omission, concerns regarding treatment, or difficulties remembering medication schedules were evaluated according to standard BMQ criteria. Consistent with validated and previously applied scoring procedures, the presence of a positive response in any one of the three domains (regimen, belief, or recall screen) was considered indicative of potential nonadherence. Participants without evidence of barriers across all domains were classified as adherent. This classification approach was used to convert domain-level responses into a binary outcome (adherent vs. nonadherent) for statistical analysis.

Operational definition

BP control was defined according to established clinical guidelines as systolic BP <140 mmHg and diastolic BP <90 mmHg. Hypertension status was categorized as controlled or uncontrolled based on the average of two standardized readings.

Study variables

Demographic and clinical variables, including age, gender, education, duration of hypertension, comorbidities, and medication adherence, were used as independent variables. The dependent variable was BP control (controlled vs. uncontrolled). Additional potential confounders, such as diabetes mellitus, the number of antihypertensive medications, and family history of hypertension, were also included in the multivariable regression model to reduce confounding bias.

Data collection tool

The study used a structured questionnaire consisting of sociodemographic variables, clinical characteristics, and the BMQ to ensure comprehensive assessment of adherence behavior and relevant covariates.

Data analysis plan

Data were entered and analyzed using Statistical Package for the Social Sciences version 25 (IBM Corp., Armonk, NY). Prior to analysis, data were screened for outliers, missing values, and normality assumptions, where applicable. Descriptive statistics, including frequencies, percentages, means, and standard deviations, were used to summarize sociodemographic and clinical characteristics of the study participants, medication adherence status, and BP control. The chi-square test was applied to assess bivariate associations between medication adherence and BP control, as well as other categorical independent variables. Binary logistic regression analysis was performed to identify factors associated with uncontrolled hypertension.

Variables with a P value of <0.20 in univariable analyses, along with variables considered clinically relevant based on previous literature, were entered into the multivariable logistic regression model. These included age, gender, education level, residence (urban/rural), duration of hypertension, diabetes mellitus, family history of hypertension, and number of antihypertensive medications. Medication adherence was included as the primary exposure variable. Multicollinearity among independent variables was assessed prior to model construction using standard diagnostic criteria to ensure model stability. Adjusted odds ratios (AORs) with 95% confidence intervals (95% CIs) were calculated to provide confounder-adjusted estimates of associations between predictors and uncontrolled hypertension. All analyses were two-tailed, and a P value of <0.05 was considered statistically significant.

Ethical considerations

Ethical approval was obtained from the institutional review boards of the participating hospitals (approval No. ATH/EC-2023/20 and 1083/DOC/KMC). Written informed consent was obtained from all participants prior to data collection. Confidentiality and anonymity were strictly maintained throughout the study. All procedures were conducted in accordance with the ethical principles of the Declaration of Helsinki for human research.

## Results

Table 1 summarizes the sociodemographic and clinical characteristics of the 436 study participants. The majority were aged 40-59 years, followed by ≥60 and 18-39 years. Male patients constituted 238 (54.59%), and female patients 198 (45.41%). Regarding education, the majority were illiterate, followed by primary education. Most participants were from urban areas. In terms of clinical profile, 178 (40.83%) had hypertension for 5-10 years, 162 (37.16%) for less than five years, and 96 (22.02%) for more than 10 years. Diabetes mellitus was present in 154 (35.32%) participants, and 176 (40.37%) reported a family history of hypertension. Additionally, 248 (56.88%) patients were on two or more antihypertensive drugs, while 188 (43.12%) were on a single drug. Medication adherence assessment showed that 196 (44.95%) patients were adherent, whereas 240 (55.05%) were nonadherent. BP was controlled in 178 (40.83%) patients and uncontrolled in 258 (59.17%).

**Table 1:** Sociodemographic, clinical characteristics, drug adherence, and blood pressure control of study participants (n = 436) HTN: hypertension; BMQ: Brief Medication Questionnaire

Variable	Category	Value, n (%)
Age (years)	18-39	68 (15.60)
	40-59	192 (44.04)
	≥60	176 (40.37)
Gender	Male	238 (54.59)
	Female	198 (45.41)
Education	Illiterate	132 (30.28)
	Primary	118 (27.06)
	Secondary	104 (23.85)
	Higher	82 (18.81)
Residence	Urban	251 (57.57)
	Rural	185 (42.43)
Duration of hypertension	<5 years	162 (37.16)
	5-10 years	178 (40.83)
	>10 years	96 (22.02)
Diabetes mellitus	Yes	154 (35.32)
	No	282 (64.68)
Family history of HTN	Yes	176 (40.37)
	No	260 (59.63)
Number of antihypertensives	1 drug	188 (43.12)
	≥2 drugs	248 (56.88)
Medication adherence (BMQ)	Adherent	196 (44.95)
	Nonadherent	240 (55.05)
Blood pressure control	Controlled	178 (40.83)
	Uncontrolled	258 (59.17)

Table 2 presents the association between medication adherence and BP control, along with bivariate associations of selected sociodemographic and clinical variables with BP control. Overall, among adherent patients (n = 196), 132 (67.35%) had controlled BP, while 64 (32.65%) had uncontrolled BP. In contrast, among nonadherent patients (n = 240), only 46 (19.17%) achieved BP control, whereas 194 (80.83%) had uncontrolled BP. This association was statistically highly significant, indicating a strong relationship between medication adherence and BP control. In addition, significant associations were observed between BP control and age group, gender, residence, duration of hypertension, presence of diabetes mellitus, and number of antihypertensive medications. Older age, longer duration of hypertension, rural residence, diabetes mellitus, and use of multiple antihypertensive drugs were all associated with poorer BP control (P < 0.05 for all comparisons).

**Table 2:** Bivariate association of medication adherence and selected clinical-demographic factors with BP control BP: blood pressure; HTN: hypertension

Variable	Category	Controlled BP, n (%)	Uncontrolled BP, n (%)	χ²	P value
Medication adherence	Adherent (n = 196)	132 (67.35)	64 (32.65)	102.84	<0.001
	Nonadherent (n = 240)	46 (19.17)	194 (80.83)		
Age (years)	18-39	42 (61.76)	26 (38.24)	34.61	<0.001
	40-59	88 (45.83)	104 (54.17)		
	≥60	48 (27.27)	128 (72.73)		
Gender	Male	102 (42.86)	136 (57.14)	18.42	<0.001
	Female	76 (38.38)	122 (61.62)		
Residence	Urban	112 (44.62)	139 (55.38)	6.91	0.009
	Rural	66 (35.68)	119 (64.32)		
Duration of HTN	<5 years	82 (50.62)	80 (49.38)	14.77	<0.001
	5-10 years	62 (34.83)	116 (65.17)		
	>10 years	34 (35.42)	62 (64.58)		
Diabetes mellitus	Yes	54 (35.06)	100 (64.94)	8.96	0.003
	No	124 (43.97)	158 (56.03)		
Number of antihypertensives	1 drug	92 (48.94)	96 (51.06)	12.33	<0.001
	≥2 drugs	86 (34.68)	162 (65.32)		

Table 3 presents the results of multivariable logistic regression analysis identifying factors associated with uncontrolled hypertension after adjustment for potential confounders. Nonadherence to medication was a strong independent predictor of uncontrolled BP, with nonadherent patients having significantly higher odds compared to adherent patients (AOR = 3.82; 95% CI: 2.45-5.97; P < 0.001). Age ≥60 years was also significantly associated with uncontrolled hypertension (AOR = 2.11; 95% CI: 1.32-3.37; P = 0.002). Diabetes mellitus (AOR = 1.76; 95% CI: 1.12-2.76; P = 0.014) and hypertension duration >10 years (AOR = 1.58; 95% CI: 1.01-2.47; P = 0.045) were also significant predictors. Although rural residence showed higher odds (AOR = 1.39; 95% CI: 0.92-2.10), this was not statistically significant (P = 0.112).

**Table 3:** Multivariable logistic regression analysis of factors associated with uncontrolled hypertension All reported associations in the multivariable model are adjusted for potential confounding variables, including age, gender, education level, residence (urban/rural), duration of hypertension, diabetes mellitus, family history of hypertension, and number of antihypertensive medications, as specified in the statistical analysis plan OR: odds ratio; CI: confidence interval; HTN: hypertension

Variable	Adjusted OR	95% CI	Wald	P value
Nonadherence to medication	3.82	2.45-5.97	36.21	<0.001
Age ≥60 years (vs. <60)	2.11	1.32-3.37	9.74	0.002
Diabetes mellitus	1.76	1.12-2.76	5.99	0.014
Duration of HTN >10 years	1.58	1.01-2.47	4.02	0.045
Rural residence	1.39	0.92-2.10	2.52	0.112
Family history of HTN	1.21	0.82-1.78	1.01	0.315
≥2 antihypertensives	1.44	0.98-2.11	3.21	0.073

## Discussion

The present study demonstrated that BP was controlled in 40.83% of participants, while 44.95% were adherent and 55.05% were nonadherent to antihypertensive therapy. These findings are consistent with national evidence from Pakistan, where adherence rates have been reported to be similarly low (approximately 36.6%), highlighting poor medication-taking behavior as a persistent challenge in hypertension management in low- and middle-income settings [16]. The slightly higher adherence observed in our study may be attributed to the inclusion of patients attending tertiary care hospitals, where access to healthcare services and clinical follow-up is comparatively better. Nevertheless, both findings reinforce that suboptimal adherence remains a major barrier to achieving adequate BP control.

A key finding of this study was the strong association between medication adherence and BP control, where 67.35% of adherent patients achieved controlled BP compared with only 19.17% of nonadherent patients (P < 0.001). This is consistent with previously published studies from Pakistan and other similar settings, which have consistently demonstrated that good medication adherence is strongly associated with improved BP outcomes and reduced cardiovascular risk [17,18]. These findings further emphasize that adherence is not merely a behavioral issue but a central determinant of therapeutic effectiveness in hypertension management.

This association was further confirmed in multivariable logistic regression analysis, where nonadherence emerged as the strongest independent predictor of uncontrolled hypertension (AOR = 3.82; P < 0.001). Similar patterns have been reported in regional studies, where poor adherence often outweighs the influence of many demographic and clinical factors in determining BP control [19,20]. This consistency across studies highlights the clinical importance of prioritizing adherence-enhancing strategies, particularly in resource-limited healthcare systems.

In addition to medication adherence, older age (≥60 years) was significantly associated with uncontrolled hypertension (AOR = 2.11; P = 0.002). This may be explained by age-related factors such as increased comorbidity burden, polypharmacy, and physiological vascular changes that contribute to more difficult BP control. Similar findings have been reported in prior studies, where elderly patients show poorer hypertension outcomes due to both biological and behavioral factors [20,21].

Diabetes mellitus was also identified as a significant predictor of uncontrolled hypertension (AOR = 1.76; P = 0.014). This finding is supported by existing literature demonstrating that the coexistence of diabetes and hypertension leads to more complex disease management and poorer cardiovascular outcomes [22]. Similarly, a longer duration of hypertension (>10 years) was associated with increased odds of poor control (AOR = 1.58; P = 0.045), possibly reflecting treatment fatigue, progressive vascular remodeling, and reduced long-term adherence.

Although rural residence was associated with higher odds of uncontrolled hypertension (AOR = 1.39), this association was not statistically significant (P = 0.112). However, this trend aligns with previous regional evidence from Khyber Pakhtunkhwa, where socioeconomic disparities, limited healthcare access, and low health literacy have been shown to negatively influence hypertension management and treatment adherence [23]. Evidence suggests that patient education, medication reminders, regimen simplification, pharmacist-led counseling, and structured follow-up programs can significantly improve antihypertensive medication adherence and BP outcomes [24,25]. Overall, these findings suggest that while adherence is the most influential factor, sociodemographic and healthcare access-related determinants also contribute meaningfully to BP control.

Strengths

This study has several notable strengths. First, the relatively large sample size enhances statistical power and improves the precision and representativeness of the findings. Second, the use of the validated BMQ strengthens the reliability and comparability of medication adherence assessment across participants. Third, BP measurements were standardized by taking two readings after adequate rest and averaging them, which minimizes measurement variability and improves the accuracy and reliability of outcome assessment. Although multivariable logistic regression was applied to adjust for potential confounders, formal model diagnostic tests such as the Hosmer-Lemeshow goodness-of-fit test, assessment of model discrimination, and detailed multicollinearity indices (e.g., variance inflation factors) were not reported. However, careful selection of clinically relevant variables and prior assessment of intervariable relationships were used to minimize the risk of model instability and confounding. Finally, the application of multivariable logistic regression analysis allowed adjustment for multiple prespecified confounding variables, thereby strengthening the internal validity of the observed associations between medication adherence and BP control.

Limitations

Despite these strengths, several limitations should be considered when interpreting the findings. First, the cross-sectional design limits the ability to establish temporal or causal relationships between medication adherence and BP control; therefore, the observed associations should be interpreted as correlational rather than causal.

Second, the use of nonprobability consecutive sampling from tertiary care hospital outpatient departments may introduce selection bias. Patients attending tertiary care centers may differ from the general hypertensive population in terms of disease severity, healthcare access, and health-seeking behavior, which limits the external validity and generalizability of the findings to community or primary care settings.

Third, medication adherence was assessed using the self-reported BMQ. Although validated, it remains subject to recall bias and social desirability bias, which may lead to misclassification of adherence status. This limitation may be particularly relevant in resource-limited settings such as Pakistan, where variations in health literacy, patient awareness, cultural perceptions, and communication with healthcare providers may further influence self-report accuracy. In addition, no objective adherence measures (such as pharmacy refill records, pill counts, electronic monitoring systems, or biochemical confirmation) were used, which could have strengthened measurement validity.

Fourth, although multivariable logistic regression was applied to adjust for confounding, residual confounding may still be present due to unmeasured variables. Important factors not included in the analysis include dietary sodium intake, physical activity level, body mass index, medication affordability, healthcare accessibility, psychosocial stress, and physician-related therapeutic inertia. The absence of these variables may have influenced the strength and precision of the observed associations.

Fifth, the hospital-based nature of the study may have resulted in overrepresentation of patients with more severe, long-standing, or poorly controlled hypertension, thereby limiting generalizability to primary care or community-based populations.

Sixth, BP control was assessed using clinic-based measurements at a single visit, which may be affected by short-term variability or the white-coat effect. Although two readings were taken and averaged to improve reliability, ambulatory or home BP monitoring was not utilized, which may have provided a more accurate assessment of true BP control.

## Conclusions

This study demonstrates that medication adherence is strongly associated with BP control among hypertensive patients attending outpatient clinics in Khyber Pakhtunkhwa, Pakistan. A markedly higher proportion of patients with controlled BP was observed among adherent patients than among nonadherent patients. In multivariable analysis, nonadherence remained a strong independent predictor of uncontrolled hypertension, along with older age, diabetes mellitus, and longer duration of hypertension. These findings indicate that poor medication adherence and specific clinical factors are significantly associated with inadequate BP control, even after adjustment for key confounders. However, due to the cross-sectional design, these findings should be interpreted as associations rather than causal relationships, as temporal direction cannot be established. The results are therefore not generalizable beyond similar outpatient hypertensive populations in comparable resource-limited settings. Nonetheless, the study provides clinically relevant evidence highlighting the importance of improving medication adherence through targeted interventions such as patient education, simplified treatment regimens, and structured follow-up strategies to improve hypertension outcomes.

## Appendices

**Table 4:** Structured study questionnaire: hypertension, medication adherence and BP control HTN: hypertension; BP: blood pressure; BMQ: Brief Medication Questionnaire; SPSS: Statistical Package for the Social Sciences

Section	Variable	Question	Response category/options	Coding (for SPSS)
A. Sociodemographic information	Age	What is your age (in years)?	Open numeric	1 = 18-39, 2 = 40-59, 3 = ≥60
	Gender	What is your gender?	Male/Female	1 = Male, 2 = Female
	Education	What is your highest level of education?	Illiterate/Primary/Secondary/Higher	1-4
	Residence	Where do you live?	Urban/Rural	1 = Urban, 2 = Rural
B. Clinical history	Duration of hypertension	How long have you been diagnosed with hypertension?	<5 years/5-10 years/>10 years	1-3
	Diabetes Mellitus	Do you have diabetes mellitus?	Yes/No	1 = Yes, 0 = No
	Family history of HTN	Do you have family history of hypertension?	Yes/No	1 = Yes, 0 = No
	Number of antihypertensive drugs	How many BP medicines are you taking?	1 drug/≥2 drugs	1-2
	Type of HTN	Diagnosis type	Primary hypertension	(Included all patients)
	Duration of treatment	Since when are you on antihypertensive medication?	Open/categorized same as HTN duration	1-3
C. Medication adherence (BMQ - regimen screen)	Missed dose	In the past week, did you miss any dose?	Yes/No	1 = Yes, 0 = No
	Dose frequency issue	Do you sometimes forget taking your medicine?	Yes/No	1 = Yes, 0 = No
	Skipping medicines	Do you ever skip your antihypertensive medication?	Yes/No	1 = Yes, 0 = No
D. BMQ - belief screen	Effectiveness belief	Do you believe your medicines control BP effectively?	Yes/No/Unsure	1-3
	Concern about side effects	Are you concerned about side effects?	Yes/No	1 = Yes, 0 = No
	Trust in medication	Do you trust your prescribed medication?	Yes/No	1 = Yes, 0 = No
E. BMQ - recall screen	Forgetfulness	Do you forget to take your medicine?	Never/Sometimes/Often	0-2
	Complex regimen	Is your medication schedule difficult?	Yes/No	1 = Yes, 0 = No
	Daily routine interruption	Does your routine affect medication intake?	Yes/No	1 = Yes, 0 = No
F. Overall medication adherence status	BMQ classification	Based on responses	Adherent/Nonadherent	1 = Adherent, 0 = Nonadherent
G. Blood pressure measurement	Systolic BP	What is your systolic BP (average of 2 readings)?	Numeric (mmHg)	Continuous
	Diastolic BP	What is your diastolic BP (average of 2 readings)?	Numeric (mmHg)	Continuous
	BP control status	Is BP controlled?	<140/90 = Controlled/≥140/90 = Uncontrolled	1 = Controlled, 0 = Uncontrolled
H. Final study variables	Independent variables	Age, gender, education, residence, duration HTN, diabetes, family history, drugs, adherence	As above	Categorical
	Dependent variable	Blood pressure control	Controlled vs. Uncontrolled	Binary outcome
